# Cellular metabolic rates and oxidative stress profiles in primary fibroblast cells isolated from virgin females, reproductively experienced females, and male Sprague‐Dawley rats

**DOI:** 10.14814/phy2.13909

**Published:** 2018-10-22

**Authors:** Joshua D. Winward, Christina M. Ragan, Ana G. Jimenez

**Affiliations:** ^1^ Department of Biology Colgate University Hamilton New York; ^2^ Department of Psychology Neuroscience Program Colgate University Hamilton New York; ^3^ Present address: Psychology Department Purdue University Northwest Westville Indiana

**Keywords:** Cellular metabolism, life‐ history, oxidative stress, reproduction

## Abstract

Life‐history theory posits that differences in reproductive strategies may dictate lifespans of organisms. Animals that have higher investments in reproduction in terms of litter size and frequency of litters tend to have shorter lifespans. The accumulation of oxidative stress damage has been proposed to be a cost of reproduction and a mediator of life‐histories among animals, however, the implications of reproduction on oxidative stress still remain unclear. We tested physiological consequences of reproduction on metabolism and oxidative stress of Sprague‐Dawley Rats (*Rattus norvegicus*) with various reproductive experiences at the cell level. We grew primary dermal fibroblasts from Sprague‐Dawley rats which have the potential of having large litters frequently. Cells were isolated from virgin females, primiparous females, multiparous females, and reproductively‐experienced males. We measured basal oxygen consumption (OCR), proton leak, ATP production, spare respiratory capacity, coupling efficiency and glycolysis using a Seahorse XF96 oxygen flux analyzer. Additionally, we measured rates of RS (reactive species) production, reduced glutathione (GSH), mitochondrial content, and lipid peroxidation (LPO) damage to quantify oxidative stress. There were no significant differences in any OCR or glycolytic parameters across any of our groups. However, reproductively‐experienced females had significantly lower rates of LPO damage as compared with virgin females and males, as well as nonsignificant decreases in GSH concentration. Decreases in LPO damage and GSH indicate that reproductively‐experienced females potentially use their endogenous antioxidant system to combat delirious effects of increased metabolism during reproduction. Our results suggest that reproduction may, in fact, have a protective effect in females.

## Introduction

Life‐history theory speculates that there are physiological and metabolic trade‐offs between reproduction and physiological maintenance associated with different evolutionary strategies (Kozlowski and Weiner 1997; Zera and Harshman 2001; Monaghan et al. 2009; Pontzer et al. 2014). The disposable soma theory of aging suggests that animals which invest heavily in allocating resources to ensure reproductive success will be unlikely to heavily invest in repair and maintenance of somatic cells (Speakman et al. 2002). When combined with life‐history theory, this theory states that if an animal “lives fast” through continuous diversion of excess energy toward reproductive effort, this animal will have a comparatively shorter lifespan than an animal which does not invest as heavily in reproduction (Pearl 1928; Speakman et al. 2002). At the population level, then, all animals can be placed on an ecological slow‐fast continuum according to the different life‐history traits they exploit. Species falling on the “slow pace of life” tend to have lower mass‐specific metabolism, slower growth trajectories, fewer offspring per year, fewer offspring per litter, and a longer lifespan (Lande 1982; Zera and Harshman 2001; Monaghan et al. 2009). Animals associated with a “fast pace of life” have a higher mass specific metabolism, faster growth trajectories, more offspring per year, more offspring per litter, and a shorter lifespan (Lande 1982; Speakman 2005; Bielby et al. 2007). Among mammals, life‐history traits scale allometrically with body size, such that larger animals are associated with slow life‐history traits whereas smaller animals are associated with fast life‐history traits (Stearns 1976; Promislow and Harvey 1990; Speakman 2005; Wiersma et al. 2007; Bielby et al. 2007; Monaghan et al. 2008). However, limited information exists about reproductive experience and changes that may accrue at the individual level and within a species. Thus, our study adds to the growing literature of cellular changes due to reproductive costs (Garratt et al. 2011; Oldakowski et al. 2012).

Reproduction, specifically physiological changes that a mother must undergo to support offspring, is a metabolically costly process (Tuomi et al. 1983; Speakman 2008; Mowry et al. 2017). These costs can be broadly divided into two different categories, physiological and ecological (Zera and Harshman 2001). Ecological costs are associated with potential increases in risk of predation with longer and more frequent foraging to sustain young. In European Kestrels (*Falco tinnunculus*), increased brood size and subsequent demand for foraging activity resulted in a higher risk of parent mortality (Daan et al. 1996). However, the most significant costs are likely associated with changes in female physiology required for two distinct processes, pregnancy and lactation. For example, during pregnancy and lactation, female rats, like many species, significantly increase their food intake, often doubling or even tripling their consumption (Speakman 2008). The corresponding morphological changes to the alimentary tract in order to process increases in food supply can increase whole‐organism metabolic costs (Speakman 2008). These changes include increases in size and length of the surface and composition of the alimentary tract to maximize the amount of nutrients that can be absorbed (Fell et al. 1963; Boyne et al. 1966). These changes to the alimentary tract, in addition to growing and feeding the fetus or multiple fetuses, increase the amount of metabolically active tissue in the mother's body. As such, total metabolic demand placed on the mother increases, but usually revert to basal levels once the mother is finished lactating, indicating that this is a plastic and metabolically expensive state that can only be sustained for brief periods of time (Randolph et al. 1977; Naya et al. 2008). Lactation specifically is an energetically demanding process for most mammalian species. For example, lactating mice demonstrate a peak increase in food intake during late lactation (Speakman 2009). Additionally, lactation highlights a cellular physiological alteration which redirects stored lipids to be synthesized in milk by the mammary glands (Hyatt et al. 2017, 2018a,b). Thus, we would expect that large litters and multiparity would place mothers in a constant state of increased metabolic demand consistent with fast life‐history traits associated with decreased longevity.

As animals produce adenosine triphosphate (ATP) through mitochondrial respiration, reactive species (RS), or pro‐oxidants, are produced as a by‐product of oxygen reduction. RS molecules contain an errant electron causing them to be highly reactive (Harman 1956). In small amounts, RS are important for cell signaling and normal cellular function (Thannickal and Fanburg 2000). However, when there is an excess of RS, they interact with other molecules, particularly lipids, to steal an electron thus generating more radicals and causing further cellular damage (Harman 1956; Hulbert et al. 2007; Monaghan et al. 2009). As lipid peroxidation (LPO) propagates across membranes, the membrane composition and function is compromised decreasing cellular function. In addition to decreases in cellular function, LPO damage instigates a complex chain reaction with highly reactive intermediaries which can propagate excess damage to proteins and DNA (Monaghan et al. 2009). Lastly as LPO increases, more energy is diverted to repair systems causing an increase in overall metabolic energy required, leading to an upregulation in metabolism (Boyd and McGuire 1991; Selman et al. 2012). During metabolically demanding processes, it is important to screen for any oxidative stress‐related effects that may occur as a result of increased metabolic and mitochondrial activity.

Animals have an endogenous defense system in the form of anti‐oxidants which can mitigate damage caused by RS (Harman 1956). Anti‐oxidants such as superoxide dismutase (SOD), catalase (CAT), vitamin E, and glutathione act by binding to RS molecules without turning into RS themselves (Monaghan et al. 2009). Thus, management of RS and anti‐oxidants becomes a balancing act that must be closely monitored to prevent the pro‐oxidant concentration from outweighing the concentration of anti‐oxidants sufficient for RS removal (Skrip and McWilliams 2016) a process known as oxidative stress. By looking for different markers of oxidative stress in animals with different reproductive and growth strategies, we can examine life history trade‐offs (Metcalfe and Monaghan 2013). If increased metabolic activity due to higher costs of female reproduction correlates with increased oxidative stress (Randolph et al. 1977; Naya et al. 2008; Monaghan et al. 2009), we would expect that females who reproduce more often would have higher rates of oxidative stress than virgin females and/or males. This would suggest that the best strategy for increased lifespan of an individual would be to not spend excess energy in reproduction, yet this has implications for the fitness of individual. Life‐history theory would suggest that organisms trade longevity of the parents to maximize number of offspring or probability of survival (Lande 1982; Speakman 2005; Monaghan et al. 2009). Thus, we would expect that having offspring may negatively affect the metabolic and oxidative stress profiles of the mother, in terms of increased energy expenditure and potential increases in oxidative damage. However, pregnancy does not seem to negatively affect mitochondrial ATP production, and may even increase the number of antioxidants produced by the individual (Schmidt and Hood 2016; Zhang and Hood 2016). This indicates that a single pregnancy itself may not be inherently detrimental to mothers, and may even provide some physiologically protective effects.

In our current study, we grew primary fibroblasts from tails of Sprague‐Dawley rats under different breeding conditions to determine differences in cellular metabolism, including basal cellular oxygen consumption (OCR), proton leak, ATP production, spare respiratory capacity, coupling efficiency, as well as glycolytic metabolic profiles, and oxidative stress profiles. Our experimental design was aimed at quantifying any differences between our groups to determine if there were any impacts on metabolic and oxidative stress profiles at the cell level that were attributed to differences in reproductive experience.

## Materials and Methods

### Animal care

All rats were housed in standard Polypropylene shoe box style cages on site at Colgate University. They all had a large scoop of hardwood chip bedding, eco bedding, a piece of cardboard bent into a tee pee shape for a shelter, and a chewable object such as a nylabone or wood block. Rats were fed Purina 5001 rat chow and water *ad libidum*. Light cycle was light/dark 12:12 h with lights on at 0900 h. with humidity kept at 30% or greater and temperature in rooms maintained at 22°C. All rats were same‐sex, pair‐housed until a week before approximate parturition date. After weaning of pups at postnatal day 21, dams were same‐sex, pair‐housed again. The multiparous females had up to 3 litters and the litters were not culled.

We were interested cellular metabolism and oxidative stress among adult reproductively ‐experienced males (*N* = 5), virgin females (*N* = 9), primiparous females (*N* = 9), and multiparous females (*N* = 5). All rats ranged in age from 364 to 368 days old. This age range was used because we have found age‐related differences in cellular metabolism in differently aged rats, thus, we wanted an aged‐matched population to isolate solely reproductive effects (Jimenez 2018). All procedures below were approved by Colgate University's IACUC committee.

### Whole animal measurements

Virgin female rats weighed 324 ± 9.8 g, whereas primiparous and multiparous rats weighed 312.87 ± 11.18 g and 324 ± 10.94 g, respectively. Male rats weighed 521.8 ± 10.28 g. Virgin female rats were 318 ± 10.85 days old at collection, whereas primiparous and multiparous rats were 344.57 ± 5.47 and 328 ± 5.22 days old at collection. Male rats were 341.6 ± 6.97 days old at collection. All primiparous and multiparous rats were collected after weaning. The days between collection and last wean date ranged between 83 and 206 days, with an average of 162 days.

### Establishment of fibroblast cell lines

We made use of tissue culture methods for two reasons. First, a nucleus from a cell isolated from skin of any species contains a nucleus with the animals’ genotype, which contains a genome of an evolutionary endpoint in the environment that the animal experiences and has undergone natural selection. Second, while respiration of isolated mitochondria can be measured, we have opted to examine the metabolic phenotypes in the whole cell as all of the regulatory elements are still intact. Dermal fibroblasts are responsible for generating connective tissue and are involved in wound healing (Sorrell and Caplan 2004), but generally this cell type is thought to be metabolically inactive until it is required at the site of tissue damage, though fibroblasts are said to be the most common cell type within an organism. However, we have found parallels in cellular metabolic rates between dermal fibroblasts and myoblasts isolated from the same animal, suggesting that dermal fibroblast are a good cell model for whole‐organism metabolism (Jimenez et al. 2014). For these reasons, these techniques may provide different insights than those available through mitochondrial isolation procedures, and could give global, cell‐level information about the metabolic and oxidative stress of the whole‐organism. Rats were sacrificed using CO_2._ We isolated primary fibroblast cells from skin of rat tails. The samples were placed in cold transfer media (Dulbecco's modified Eagle medium [DMEM], with 4500 mg/L glucose, sodium pyruvate, and 4 mmol/L L‐glutamine supplemented with 10% heat‐inactivated fetal bovine serum, and antibiotics [100 U/mL pen/strep], containing 10 mmol/L HEPES) until processing. To isolate primary fibroblast cells, skin samples were sterilized in 70% ethanol and 20% bleach. Skin was minced and incubated in sterile 0.5% Collagenase B overnight in an atmosphere of 37°C 5% CO_2_ and 5% O_2_. After incubation, the collagenase mixture was filtered through a sterile mesh (Falcon No. 352340), and centrifuged at × 1000 rpm for 5 min. The resulting supernatant was removed, and the pellet was resuspended with 7 mL of mammal media (Dulbecco's modified Eagle medium [DMEM], with 4500 mg/L glucose, sodium pyruvate, and 4 mmol/L L‐glutamine supplemented with 10% heat‐inactivated fetal bovine serum, and antibiotics [100 U/mL pen/strep]). Cells were grown in culture flasks at 37°C in an atmosphere of 5% O_2_/CO_2_. When cells reached 90% confluence, they were trypsinized (0.05%) and cryopreserved at 10^6^ cells/mL in DMEM supplemented with 40% fetal bovine serum and dimethylsulfoxide (DMSO) at a final concentration of 10%. We stored cells in liquid N_2_ prior to assessment of their cellular metabolism and oxidative stress profile (below). All measurements were conducted using cells at passage 2 (P2).

### Metabolic profiles

A Seahorse XF‐96 Extracellular flux analyzer was used to measure the rate of O_2_ consumption and glycolysis in isolated primary dermal fibroblast cells from all rats. Assays were performed prior to experiments to determine the optimal cell seeding density, and optimal concentrations of each compound used. We seeded 20,000 cells per well in duplicate per individual into XF‐96 cell culture plates and allowed cells to attach overnight.

### Oxygen consumption rates (OCRs)

OCR was determined using XF‐96 FluxPaks (37°C) from Seahorse Bioscience. We measured OCRs after cells were equilibrated to running media for 1 h, which contained 10 mmol/L glucose, 1 mmol/L sodium pyruvate and 2 mmol/L glutamine, pH = 7.4. Baseline measurements of OCRs were made three times prior to injecting a final well concentration of 2 μmol/L oligomycin, which inhibits ATP synthesis by blocking the proton channel of the Fo portion of the ATP synthase. This method can be used to distinguish the percentage of O_2_ consumption devoted to ATP synthesis and the O_2_ consumption required to overcome the natural proton leak across the inner mitochondrial membrane plus any non‐mitochondrial O_2_ consumption. Next, we injected a final well concentration of 0.125 μmol/L FCCP, which is an uncoupling agent that disrupts ATP synthesis by essentially collapsing the proton gradient across the mitochondrial membrane leading to uncoupled consumption of energy and O_2_ without generating ATP: this provides a theoretical maximal respiratory rate. Finally, we injected a final well concentration of 0.5 μmol/L Antimycin A, a Complex III inhibitor and rotenone, a Complex I inhibitor. This combination stops mitochondrial respiration and enables non‐mitochondrial respiration to be evaluated (Gerencser et al. 2009; Brand and Nicholls 2011; Rogers et al. 2011; Hill et al. 2012).

After measurements were completed, we used a Countess II FL cell counter to count the actual final concentration of cells in each well and normalized all rates to a total of 20,000 cells. For each OCR parameter, we followed the equations supplied by Divakaruni et al. (2014). Spare respiratory capacity is calculated by the difference between basal respiration and FCCP‐induced maximal respiration, and this measurement describes the cell's capability to respond to periods of stress or high ATP demand. ATP production is the difference between basal respiration, and oligomycin sensitive respiration, and this measurement describes the ATP demand of the cell (Divakaruni et al. 2014). Coupling efficiency is the ratio of ATP production and basal respiration.

### Extracellular acidification rate (ECAR)

ECAR values were measured in units of mpH, which is the change in pH in the media surrounding the cells due to proton flux in glycolysis. Measurements of ECAR were performed after the cells were equilibrated to running media for 1 h. Running media contained no glucose and 2 mmol/L L‐glutamine in all experiments, pH = 7.4. Baseline rates were measured three times prior to any injections. We first injected a final well concentration of 10 mmol/L glucose into media surrounding cells, which provides a measure of glycolytic rate, and then we injected a final well concentration of 2 μmol/L oligomycin, giving us an estimate of glycolytic capacity in cells. Finally, we injected a final well concentration of 50 mmol/L 2‐DG, a glucose analog that inhibits glycolysis, which provided an estimate of non‐glycolytic acidification (Hill et al. 2012). After measurements were completed, we used a Countess II FL cell counter to count the actual final concentration of cells in each well and normalized all rates to a total of 20,000 cells. For each ECAR parameter, we followed the equations supplied by Divakaruni et al. (2014).

### Oxidative stress profiles

For oxidative stress measurements, cells were seeded at 10,000 cells per well and allowed to attach for 24 h prior to any experiments. We used Thermo Scientific™ Nunc™ MicroWell™ 96‐Well Optical‐bottom black chimney plates with polymer base treated with ply‐d‐lysine for all fluorescent stains. After staining with each fluorescent stain (below) on a separate plate, cells were imaged using a Tecan infinite measure m200 fluorescent plate reader. Due to differing growth rates of each cell line, cells were counted after each experiment using a Countess II FL cell counter and data were normalized to a total of 20,000 cells per well.

### Reduced glutathione (GSH)

ThiolTracker™ Violet Kit (Glutathione Detection Reagent, Molecular Probes^®^) was used to measure concentration of reduced glutathione (GSH). GSH reduces the oxidized form of the enzyme GPx, which in turn reduces H_2_O_2_. Cells were rinsed with sterile PBS twice and 20 μM ThiolTracker was added to each well. Plates were incubated at 37°C 5% CO_2_/O_2_ for 30 min. Cells were thenwashed another three times with sterile PBS, and imaged in phenol red‐free FluoroBrite DMEM. Excitation and emission were measured in the violet spectrum at 404/526 nm, respectively (Mandavilli and Janes 2010; Roberts et al. 2017). We used a concentration of 1–50 μmol/L Glutathione (Sigma cat no. G2451) as a standard of comparison while using this probe with our cells.

### RS production

CellROX ^®^ Green Oxidative Stress Reagents kit (Molecular Probes ^®^) was used to measure RS production. CellROX reagents were added directly to the serum‐free medium at a concentration of 5 μmol/L to cells and incubated at 37°C for 30 min. Cells were then washed three times with sterile PBS, and then imaged in phenol red‐free FluoroBrite DMEM. Excitation and emission were in the green spectrum at 488/530 nm, respectively (Gebhard et al. 2013; Choi et al. 2015). As a positive control, cells were treated with or without 100 μmol/L manadione (Sigma cat no. M5625) prior to being stained with CellROX, to confirm that the signal was due to ROS production.

### Mitochondrial content

Mitochondrial content was measured using MitoTracker^®^ Mitochondrion‐Selective Probes (Molecular Probes ^®^). Cells were stained with a 20 nmol/L of MitoTracker Deep‐Red and incubated at 37°C 5% CO_2_/O_2_ for 60 min. Cells were then, washed three times with sterile PBS and imaged in phenol red‐free FluoroBrite DMEM. MitoTracker^®^ excitation and emission were in the red spectrum at 635/670 nm (Miyake et al. 2011; Jimenez et al. 2014).

### Lipid peroxidation damage

Lipid peroxidation (LPO) was measured with the Image‐iT^®^ Lipid Peroxidation Kit based on the BODIPY^®^ 581⁄591 reagent The ratio between red to green indicates the degree of lipid peroxidation. Cells were stained with 10 μmol/L of component A and incubated at 37°C 5% CO_2_/O_2_ for 30 min. Cells were then washed three times with sterile PBS and imaged in phenol red‐free FluoroBrite DMEM. LPO red excitation and emission were 575/610 nm, respectively, and LPO green was 488/525, respectively (Leirós et al., 2014; Dezest et al. 2017). A final concentration of 100 μmol/L Cumene hydroperoxide was used as a positive control to induce lipid peroxidation.

### Statistics

Data from every assay were first tested for normality using a Kolmogorov‐Smirnov test. If not normal, the data were log‐transformed and re‐tested for normality prior to other statistical analyses to meet assumptions of an ANOVA. For this study, we log‐transformed proton leak, coupling efficiency, glycolysis, reduced glutathione, RS production, mitochondrial content, and LPO damage data sets prior analyses. These data were then analysed using a two‐way ANOVA, accounting for sex and differences in number of litters, primiparous or multiparous. Results were considered significant if *P* < 0.05. All statistical tests were performed using SPSS 24.0.

## Results

### Oxygen consumption rates (OCRs)

**Figure 1:**
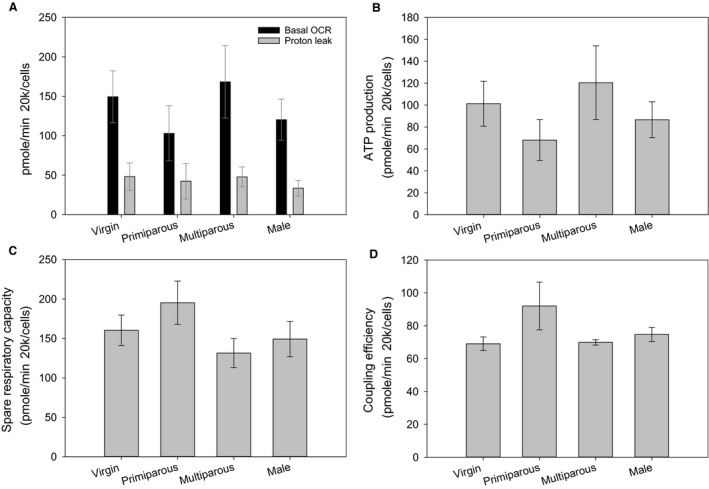
Differences in OCR parameters between males, virgin females, primiparous females, and multiparous females for (A) proton leak and basal oxygen consumption; (B) ATP production; (C) spare respiratory capacity; (D) coupling efficiency. For all parameters samples sizes were males (*N* = 5), virgin females (*N* = 9), primiparous females (*N* = 9) and multiparous females (*N* = 5). Values are presented as averages ± SEM. Asterisk (*) highlight significant differences.

We found no significant differences in any OCR parameters with respect to sex (Basal OCR *F* = 0.132, *P* = 0.720; Proton leak *F* = 0.144, *P* = 0.707; ATP production *F* = 0.052, *P* = 0.822; Spare respiratory capacity *F* = 0.187, *P* = 0.669; Coupling efficiency *F* = 0.263, *P* = 0.612; Fig. 1).

We found no differences in any OCR parameters between virgin females and primi‐ or multiparous females (Basal OCR *F* = 0.807, *P* = 0.458; Proton leak *F* = 0.841, *P* = 0.444; ATP production *F* = 1.381, *P* = 0.271; Spare respiratory capacity *F* = 1.248, *P* = 0.305; Coupling efficiency *F* = 0.009, *P* = 0.991; Fig. 1).

### Extracellular acidification rate (ECAR)

**Figure 2:**
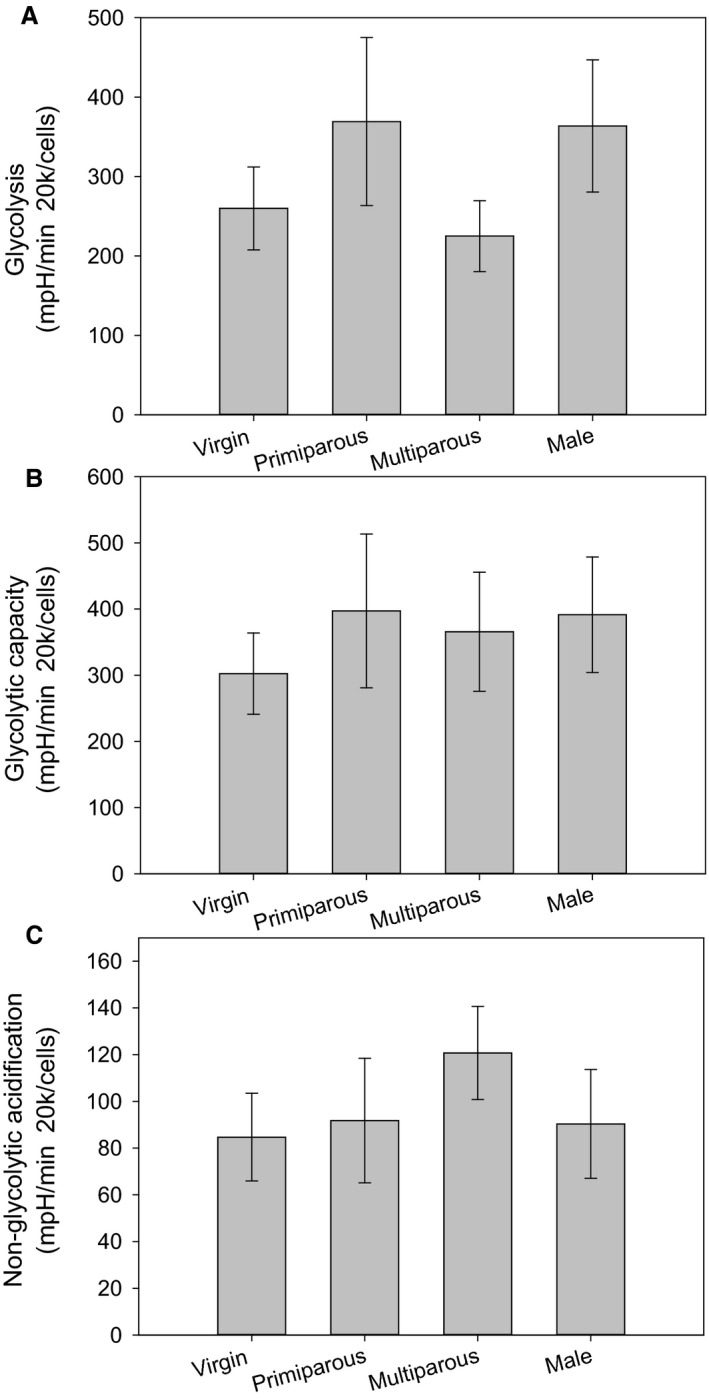
Differences in ECAR parameters between males, virgin females, primiparous females, and multiparous females for (A) Glycolysis; (B) Glycolytic capacity; (C) Non‐glycolytic acidification. For all parameters samples sizes were males (*N* = 5), virgin females (*N* = 9), primiparous females (*N* = 9) and multiparous females (*N* = 5). Values are presented as averages ± SEM. Asterisk (*) highlight significant differences.

We found no significant differences in any ECAR parameters with respect to sex (Glycolysis *F* = 0.675, *P* = 0.419; Glycolytic capacity *F* = 0.123, *P* = 0.729; Non‐glycolytic acidification *F* = 0.030, *P* = 0.865; Fig. 2).

We found no significant differences in any ECAR parameters between virgin females and primi or multiparous females (Glycolysis *F* = 0.085, *P* = 0.919; Glycolytic capacity *F* = 0.166, *P* = 0.848; Non‐glycolytic acidification *F* = 0.621, *P* = 0.546; Fig. 2).

### Oxidative stress profiles

**Figure 3:**
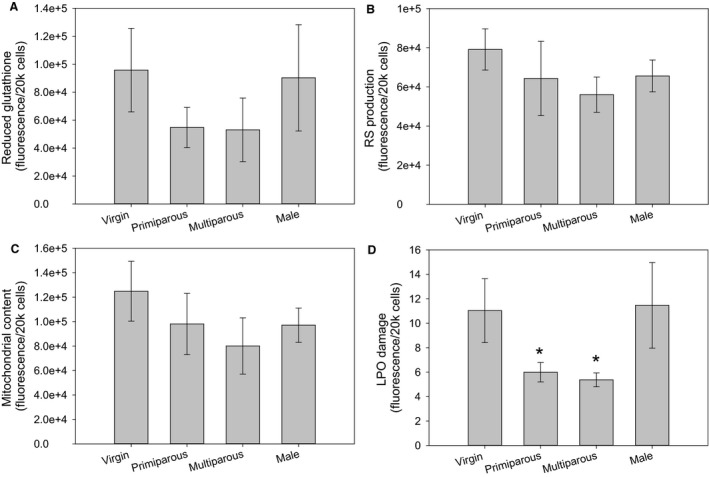
Differences in measurements of oxidative stress between males, virgin females, primiparous females, and multiparous females for (A) Reduced Glutathione; (B) RS production; (C) Mitochondrial content; (D) Lipid Peroxidation. For all parameters samples sizes were males (*N* = 5), virgin females (*N* = 9), primiparous females (*N* = 9) and multiparous females (*N* = 5). Values are presented as averages ± SEM. Asterisk (*) highlight significant differences.

We found no significant differences in reduced glutathione (*F* = 0.203, *P* = 0.656), RS production (*F* = 0.066, *P* = 0.799), mitochondrial content (*F* = 0.003, *P* = 0.954), and LPO damage (*F* = 2.135, *P* = 0.156) between males and females (Fig. 3).

We found no significant differences in reduced glutathione (*F* = 0.596, *P* = 0.559), RS production (*F* = 1.864, *P* = 0.177), mitochondrial content (*F* = 1.203, *P* = 0.318) across between virgin females and primi or multiparous females, however, we did see significant differences in LPO damage (*F* = 5.583, *P* = 0.010) with reproduction (Fig. 3), wherein reproductively active females, primiparous and multiparous, had lower LPO compared with virgin females and males.

## Discussion

Life‐history trade‐offs are mediated by physiological and ecological mechanisms (Speakman 2008). Both mechanisms have an inherent cost on the fitness of the organism; the costliest of these processes are the physiological impacts that pregnancy and lactation have on mothers (Tuomi et al. 1983; Speakman 2008; Mowry et al. 2017). During reproduction, mothers must alter their physiology to accommodate the increased demand for nutrients to support their offspring (Kennedy et al. 1958; Fell et al. 1963; Boyne et al. 1966; Jolicoeur et al. 1980; Speakman 2008). With this increased nutritional demand from the offspring comes a concomitant increase in metabolic demand on the mother, thus, mothers increase the total amount of metabolically active tissue in order to support these changes (Speakman 2008). Such an upregulation of metabolically active tissue can be accompanied by an increase in the production of harmful RS (Randolph et al. 1977; Naya et al. 2008). Thus, reproduction in mammals is a period of high reproductive plasticity (Hood et al. 2018), with physiological changes that can alter a female's physiology for the remainder of her lifespan (Zhang and Hood 2016). We found that the primiparous and multiparous females showed no differences with respect to oxygen consumption and glycolytic rates compared with virgin females and had significantly lower rates of oxidative damage which was likely facilitated by the utilization of their endogenous antioxidant reserves. We expected that reproduction would essentially alter baseline conditions, so that collecting samples months after last weaning may demonstrate different baselines between virgins and reproductively experienced females.

Across all groups, there was no significant difference in any of the OCR or ECAR parameters despite our different reproductive treatments. Whole‐animal metabolic rates in rats were consistent with our cellular metabolic rates, such that the act of reproduction does not significantly alter baseline oxygen consumption. (Trojan and Wojciechowska 1967; Dryden et al. 1974; Randolph et al. 1977; Studier 1979; McClure and Randolph 1980; Mattingly and McClure 1982; Nicoll and Thompson 1987; Prentice and Whitehead 1987; Rose 1987; Weiner 1987). This lack of metabolic difference is likely due to the fact that all of the samples were collected post‐lactation, although it is interesting that we did not observe a change in baseline conditions across treatments as others have found (Zhang and Hood 2016). Whereas the effects of reproduction on glycolytic pathways are sparse, there is some evidence that suggests that during and immediately after parturition there is a marked increase in glycolysis (Sutton and Pollak 1980). However to our knowledge, the effects of different reproductive states on rates of glycolysis after parturition and lactation have not been measured before. This lack of change in oxygen consumption and glycolysis is likely due to the fact that although pregnant and lactating rats have higher amounts of metabolically active tissue, and increases in mammary glands for lactation, their daily locomotor activity significantly decreases during this process (Slonaker 1924; Randolph et al. 1977).

Our results for cellular oxidative stress did not support the hypothesis that reproduction increases the amount of oxidative stress experienced by post‐reproductive females. Instead, we found that rates of LPO damage were significantly lower in cells isolated from primiparous and multiparous females when compared with cells isolated from virgin females and males. None of our other parameters for oxidative stress were significantly different across groups. Although, it is noteworthy to point out, that we saw a nonsignificant decrease in reduced glutathione (GSH) concentration in cells from primiparous and multiparous females when compared with the other groups. GSH is the oxidized version of the enzyme GPx which has been shown to directly reduce concentrations of LPO damage (Halliwell and Chirico 1993; Catalá 2009; Ayala et al. 2014). GPx reduces H_2_O_2_, or organic hydrogen peroxide and harmful RS molecule, to water and alcohol and is one of the only enzymes that directly prevents the cascade of LPO damage caused by H_2_O_2_ (Ayala et al. 2014). We also found that RS production remains constant across treatment groups, thus, decreases in GSH concentration can be attributed to post‐reproductive females using their antioxidant reserves to combat LPO damage during reproduction, while RS production does not increase with increases in metabolic demand (Metcalfe and Monaghan 2013; Speakman and Garratt 2014).

To our knowledge, this is the first study examining reproductive effects at the cell level on primary fibroblast cells. Previous work has found differences between muscle (post‐mitotic) and liver (mitotic) tissues during reproduction. Specifically, the capacity for mitochondrial respiration increased in liver mitochondria from animals that lactated compared with those who did not, even after 12 weeks from weaning of pups, whereas muscle mitochondria showed a decrease in capacity for respiration in the same groups (Hyatt et al. 2017). These differences may stem from the mitotic differences between these tissues, as previously found by others (Harper et al. 2004; for example). It is noteworthy to point out that in previous work highlighted above, it is the mitotically active tissue that expresses a difference with respect to reproduction. Fibroblast cells are also mitotically active, thus, may render informative for the whole‐animal phenotype. Other studies on small rodents have shown similar results when measuring oxidative damage as well as antioxidant capacity. In Bank Vole (*Myodes gareolus*) females, oxidative damage was measured by quantifying Malondialdehyde (MDA) formation, which is often used as a marker for LPO concentration. Similar to the results of our study, MDA concentrations were significantly lower in skeletal and kidney tissues of reproducing females, and there was no difference in LPO damage between the primiparous and multiparous females, indicating a decrease in oxidative damage as a result of reproduction (Oldakowski et al. 2012). Additionally, in house mice (*Mus musculus domesticus*), oxidative stress was quantified in blood, liver, and gastrocnemius muscle tissue by measuring MDA, glutathione, and protein thiol concentrations using chromatography, and recycling assays. Similar to our findings, there were decreases in the LPO damage found in blood and liver tissue in females that were allowed to reproduce *ad libidium* (Garratt et al. 2011). However, glutathione concentration in lactating and pregnant individuals was found to increase or remain constant, this is most likely due to the effect of estradiol and is impact on glutathione as discussed below. Even in a study examining female lizards, four separate RS (singlet oxygen, peroxynitrite, superoxide and H_2_O_2_), were quantified in blood tissue. When levels of these RS were measured after the lizards had laid their eggs, levels of RS were significantly decreased in females that had a relatively higher reproductive effort in the form of larger clutch sizes (Olsson et al. 2009). These studies, as well as our own, are indicative of a growing base of research that has found decreases in markers for oxidative damage in reproductively experienced females (Olsson et al. 2009; Alonso‐Alvarez et al. 2004; Beaulieu et al. 2011; Bergeron et al. 2011; Garratt et al. 2011; Isaksson et al. 2011; Markó et al. 2011; Garratt et al. 2012; Oldakowski et al. 2012; Wilson et al. 2012; Metcalfe and Monaghan 2013).

Although recently, the literature seems to be moving toward the conclusion that reproduction does not have a negative effect on the amount of oxidative damaged accrued by females, there seems to be less of a clear conclusion as to why this is the case (Olsson et al. 2009; Alonso‐Alvarez et al. 2004; Beaulieu et al. 2011; Bergeron et al. 2011; Garratt et al. 2011; Isaksson et al. 2011; Markó et al. 2011; Garratt et al. 2012; Oldakowski et al. 2012; Wilson et al. 2012; Metcalfe and Monaghan 2013). In the previous studies mentioned above, both pro‐oxidant profiles as well as anti‐oxidant profiles from blood, liver, and skeletal muscle tissue show similar results for LPO damage to our studies, however, all of these samples were taken from post‐reproductive females immediately after parturition and during lactation (Garratt et al. 2011; Oldakowski et al. 2012; Mowry et al. 2016). In these studies, the authors propose that the antioxidant concentration is upregulated in order to compensate for increases in metabolic rate and potential increases in RS, thus, decreasing LPO damage. These results are in contrast to our findings, likely because our reproductively experienced females were past parturition and lactation. Instead of finding any increases in GSH, we found a non‐significant decrease in GSH in post‐reproductive females as compared with non‐reproductive females and males. There are two main differences between our study and those that report increases in antioxidant concentration which may explain this discrepancy. First, our rats were all sacrificed and samples analyzed after females had completed lactation. In previous studies, measurements for antioxidant concentration were taken immediately after parturition or during lactation (Garratt et al. 2011; Oldakowski et al. 2012; Mowry et al. 2016). Secondly, there is a link between estradiol concentrations and increases in glutathione. In ovariectomized rats, those that were supplemented with estradiol produced significantly more glutathione than those that were not supplemented with estradiol, and those that were not supplemented with estradiol accrued significantly more LPO damage than those that were supplemented (Borrás et al. 2003; Kireev et al. 2007). Estradiol acts by upregulating the production of glutathione molecules, thereby, mitigating the effects of any potential oxidative damage (Sack et al. 1994; Arnal et al. 1996; Sudoh et al. 2001; Borrás et al. 2003; Kireev et al. 2007)**.** In mammals, estradiol reaches a peak immediately after parturition, thus, any samples taken near parturition may demonstrate increases in glutathione as well (McCormack and Greenwald 1974; Cushing et al. 1995). Because our samples were taken after lactation was complete, animals tested here may have been required to deplete their glutathione reserves to combat the LPO damage without having the benefit of increased estradiol to replenish them.

Our study helps to add to the growing body of research that states that there is a protective effect of reproduction in fast‐paced animals by reducing the amount of oxidative damage in reproductively experience females (Olsson et al. 2009; Alonso‐Alvarez et al. 2004; Beaulieu et al. 2011; Bergeron et al. 2011; Garratt et al. 2011; Isaksson et al. 2011; Markó et al. 2011; Garratt et al. 2012; Oldakowski et al. 2012; Wilson et al. 2012; Metcalfe and Monaghan 2013). Our paper in the context of current literature shows the importance of treating life‐history as a dynamic issue, and that it is not simply an energetics trade‐off, but that reproductive strategies, metabolic and oxidative profiles, and timing of sample collection must be considered in concert to gain a more complete understanding of the causes of life‐history theory.

## Conflict of Interest

The authors have no conflict of interests to report.
